# Ensemble Methods with Voting Protocols Exhibit Superior Performance for Predicting Cancer Clinical Endpoints and Providing More Complete Coverage of Disease-Related Genes

**DOI:** 10.1155/2018/8124950

**Published:** 2018-01-10

**Authors:** Runyu Jing, Yu Liang, Yi Ran, Shengzhong Feng, Yanjie Wei, Li He

**Affiliations:** ^1^Shenzhen Institutes of Advanced Technology, Chinese Academy of Sciences, Shenzhen 518055, China; ^2^College of Chemistry, Sichuan University, Chengdu 610064, China; ^3^Biogas Appliance Quality Supervision and Inspection Center, Biogas Institute of Ministry of Agriculture, Chengdu, Sichuan, China

## Abstract

In genetic data modeling, the use of a limited number of samples for modeling and predicting, especially well below the attribute number, is difficult due to the enormous number of genes detected by a sequencing platform. In addition, many studies commonly use machine learning methods to evaluate genetic datasets to identify potential disease-related genes and drug targets, but to the best of our knowledge, the information associated with the selected gene set was not thoroughly elucidated in previous studies. To identify a relatively stable scheme for modeling limited samples in the gene datasets and reveal the information that they contain, the present study first evaluated the performance of a series of modeling approaches for predicting clinical endpoints of cancer and later integrated the results using various voting protocols. As a result, we proposed a relatively stable scheme that used a set of methods with an ensemble algorithm. Our findings indicated that the ensemble methodologies are more reliable for predicting cancer prognoses than single machine learning algorithms as well as for gene function evaluating. The ensemble methodologies provide a more complete coverage of relevant genes, which can facilitate the exploration of cancer mechanisms and the identification of potential drug targets.

## 1. Introduction

With the development of genetic sequencing technology, genetic information could be recorded as gene expression data. Data mining, such as using machine learning methods, is commonly used to reveal latent correlations between diseases and gene expression. Supervised or semisupervised machine learning algorithms have been proposed to predict the clinical outcomes of cancers [1–4] in the context of tumorigenesis. The support vector machine (SVM) [5–8] and artificial neural network (ANN) [9–11] algorithms were the most commonly used approaches for predicting prognoses. In addition, the Bayesian probability model [12–14] and the fuzzy neural network [15] were also used for cancer prognosis prediction. The microarray quality control (MAQC) project thoroughly investigated the performance of models for the prediction of clinical outcomes of breast cancer, multiple myeloma, and neuroblastoma and were common practices for microarray-based model construction and validation [16]. The network-based approaches have seen a recent widespread use for the identification of cancer-related genes and have revealed the molecular mechanisms of various cancers [17–22]. However, to the best of our knowledge, no studies have examined multiple algorithms with two different kinds of expression data and their ensemble performance with a limited number of samples, which might be crucial when using them in practical. In most cases, the number of available samples is restricted due to the cost, privacy, and other reasons. A limited sample number causes a predictive model to be more sensitive to the dataset distribution, and the lack of prior knowledge simultaneously reduces the overall predicting performance which could be reflected by MCC (Matthews correlation coefficient). Moreover, a single machine learning algorithm provides insufficient coverage of the disease-related genes because it only uses genes that show the greatest difference of expression profiles between the phenotypic statuses compared when the genes have a similar function. Thus, identifying a stable predictive model using a limited number of samples becomes a challenge.

To address this problem, the present study thoroughly investigated the performance of single models among different datasets and proposed a strategy to combine multiple predictive models as well as the datasets into a final ensemble for clinical prediction. Compared with a single machine learning algorithm, an ensemble scheme could not only perform more reliably when predicting clinical endpoints but could also provide broader coverage of disease-related genes, which will be beneficial for further downstream analysis in such applications as the identification of potential drugs.

## 2. Materials and Methods

**Figure 1:**
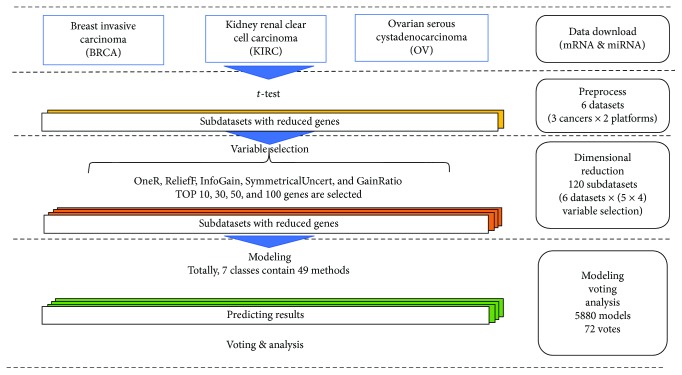
Work flow of the whole process. First, the datasets were downloaded from the GDC (Genomic Data Commons) database. Next, the downloaded mRNA and microRNA sequencing data are united by the usable information. The *t*-test was used afterwards to determine the significantly expressed genes. Five selection methods were used to select the cancer-associated genes and the subdatasets generated according to the ranks. Finally, the prediction results were integrated by a voting protocol. Note that every subdataset was divided into two pieces for cross-validation and independent test in the ratio 4 : 1 before variable selection. Only the datasets for cross-validation will be used for variable selection and modeling.

The workflow of this study is listed in Figure 1. The datasets were carefully generated such that the scale and representation of the samples in the different datasets are consistent such that the predictions were comparable. This section describes the data and methods used.

### 2.1. GDC Data

All data were downloaded from the NCI's Genomic Data Commons (GDC) [23] by using the official web-based API (https://gdc.cancer.gov/developers/gdc-application-programming-interface-api). The genomic data were from the official normalized microRNA and RNA sequence expression data because it was restricted by multiplatform coverage and accessibility; a portion of the cancer data was excluded from this study. For example, the number of available samples of neuroblastoma in GDC is 1127 (this number might change if the database is updated), but only approximately half of these samples have associated RNA sequence data, and no microRNA sequence data are available. Finally, the freely accessible data for breast invasive carcinoma (BRCA), ovarian serous cystadenocarcinoma (OV), and kidney renal clear cell carcinoma (KIRC) were downloaded and used for modeling. The clinical information was downloaded in the XML format. The relationships of samples from different platforms (such as mRNA, microRNA, and clinical) were identified by the official MetaData file in the JSON format. The detailed distribution can be found in Table 1.

### 2.2. Preprocessing

Since we wanted to make the results from two sequencing platforms (e.g., mRNA and microRNA) comparable and able for voting at last, the selection of samples was determined by the integration of different platforms. Only samples that contained clinical information and expression data for both mRNA and microRNA were retained for subsequent analysis.

The sample label was determined by the clinical information. For OV and KIRC, the label was determined by the survival time. A sample was deemed positive if the recorded survival time was larger than one year and if the patient was still alive (based on the clinical data). Similarly, a sample was deemed negative if the associated patient was dead and if the recorded survival time was less than one year. The sample label for BRCA was determined by the estrogen receptor (ER) status (negative or positive), which was also recorded in the clinical XML file. Therefore, if the required information for a sample could not be found in the clinical data or its status did not satisfy the criteria (e.g., the patient was alive but the survival time was less than one year), it was excluded.

The sample number was reduced by the clinical information. Student's *t*-test was used to subsequently reduce the mRNA and microRNA numbers. Only genes that had significant expression with a *p* value less than 0.05 were retained in a dataset. The ratio of positive to negative samples was kept in an appropriate range to reduce classification bias. In this study, the range of this ratio was 0.5 to 2. For example, if a dataset contained 22 positive and 50 negative samples, 6 negative samples were randomly removed to adjust the ratio so it fell within the required range. The eliminated datasets were divided into two parts for cross-validation and independent tests in a 4 : 1 ratio. The scale of the datasets is listed in Table 1. Only the datasets for cross-validation will be used for variable selection and the 5-fold cross-validation; the datasets for independent testing will not participate in modeling. And only the independent prediction will be used for further ensemble analysis and comparison.

### 2.3. Machine Learning Methods

49 modeling methods in WEKA [24] (version 3.8.1) were investigated in this study. The methods were divided into seven different classes by the developers of WEKA according to specific features of the methods (Table 2). The different method classes had different features. In the *functions* class, most of the methods use a functional solution for modeling the data, such as *LibSVM* [25] and logistic regression [26], and in most cases, few mechanisms are available for ensemble learning, such as voting or resampling. However, in the *meta* class, the methods use resampling and voting for classification and regression, and the methods in the other classes are considered model units, such as *AdaBoost* [27] and *Bagging* [28]. The methods from the *bayes* class are from the probability and graph theory, and most of them, including *NaïveBayes* [29], *BayesNetwork* [30], and *BayesianLogisticRegression* [31], are sensitive to sample number. Similarly, methods in the *rules* class use rules (such as decision table) for classification [32]. The methods in the *lazy* class are instance-based and could be optimized for better efficiency using a lazy algorithm [33]. Most of the methods in the *trees* class are based on the classification and regression tree algorithm, but the way they are carried out is different. Many other mechanisms are integrated into the *trees* such as resampling used in a random forest [34]. Finally, the *misc* class contains methods for which it is difficult to assign to another class. Only two methods fell into this class in this study, namely, the *VFI*, an ensemble method based on a voting protocol [35], and the *HyperPipes*, based on an algorithm that finds similarities among attributes. Considering running time, comparability, and reducing the risk of overfitting, only default parameters are used for modeling.

Because the sample number was limited, fewer genes should be considered to avoid overfitting. In this study, five variable selection methods were used for dimensional reduction: *OneR*, *ReliefF*, *InfoGain*, *SymmetricalUncert*, and *GainRatio*. *OneR* is executed by using the *OneR* classifier, which is based on measuring the error between the attributes and the response values [36]. *ReliefF* uses a resampling mechanism for evaluating the attributes [37]. The other methods are from the information theory, and the associated formulas are

(1) $$\begin{array}{c} GainRatio=\left(class,attribute\right)=\frac{H\left(class\right)-H\left(class\;|\;attribute\right)}{H\left(attribute\right)},\\ InfoGain\left(class,attribute\right)=H\left(class\right)-H\left(class\;|\;attribute\right),\\ SymmetricalUncert\left(class,attribute\right)=2\frac{H\left(class\right)-H\left(class\;|\;attribute\right)}{H\left(class\right)}+H\left(attribute\right), \end{array}$$

where the “*H*()” in the formula is the information entropy (Shannon entropy) [38] and “class” denotes the values of a label. According to their formulas, *GainRatio* could be considered as a normalization of *InfoGain*. However, the information entropies of all of the attributes, such as the expression of mRNAs and microRNAs, are different, so both methods are used in this study. By using the ranking mechanism in WEKA, in every subdataset for cross-validation, the attributes can be ranked, and the top 10, 30, 50, and 100 ranked attributes are selected for cross-validation and modeling. Note that since the number of microRNAs in OV is limited (83, which is less than 100), the numbers of the attributes in the subdatasets are 10, 30, 50, and 83. Totally, we investigated a total of 5 variable selection methods × 4 subdatasets × 49 modeling methods = 980 predictive models.

### 2.4. Integrating the Predictions by Voting

After generating hundreds of models, it is possible to combine their predictions. As previously mentioned, the prediction performances are ranged in the datasets. To integrate the ranged predictions and find a stable modeling method for genetic datasets, we used a voting protocol in this study to identify the datasets.

All of the weights were the same, except in the information theory methods such as *InfoGain*, *SymmetricalUncert*, and *GainRatio*. The weights in the information theory methods were modified to 1/3 when voting the predictions from the subdatasets of the OV-miRNA group according to the prediction distribution. More details may be found in “the coverage and reliability of selected genes” and “the distributions of the predictions.”

Three voting schemes were used to arrive at a comprehensive conclusion. All of the methods were first used for voting. Then, some of the methods which performed poorly for all datasets were eliminated. Finally, mRNA and miRNA datasets were combined for voting.

### 2.5. Measurement Methods

Since there were many predictions, box plots were used to reflect the stability of the different classes. The quartiles Q1 and Q3, the interquartile range (IQR), and the whiskers (the lower whisker is Q1 −1.5 IQR, and upper whisker is Q3 +1.5 IQR) in the box plot are discussed. Two types of box plots were used to present the results in different angles, one based on the WEKA classes of modeling methods and another based on the scale of the subdatasets. Because the ratio of positive to negative samples was biased, the Matthews correlation coefficient (MCC) was used as the criterion for the plots. The MCC is one of the criteria used to evaluate the prediction performance, and the associated formula is

(2) $$\begin{array}{c} MCC=\frac{TP\times TN-FP\times FN}{\sqrt{\left(TP+FN\right)\times \left(TP+FP\right)}\times \left(TN+FP\right)\times \left(TN+FN\right)}, \end{array}$$

where TP, TN, FP, and FN are the number of true-positive predictions, true-negative predictions, false-positive predictions, and false-negative predictions, respectively.

## 3. Results

**Figure 2:**
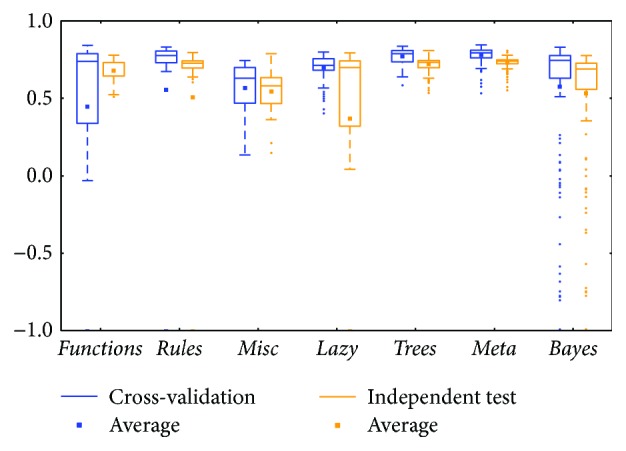
MCC of the BRCA-mRNA group by the *functions* class.

**Figure 3 fig3:**
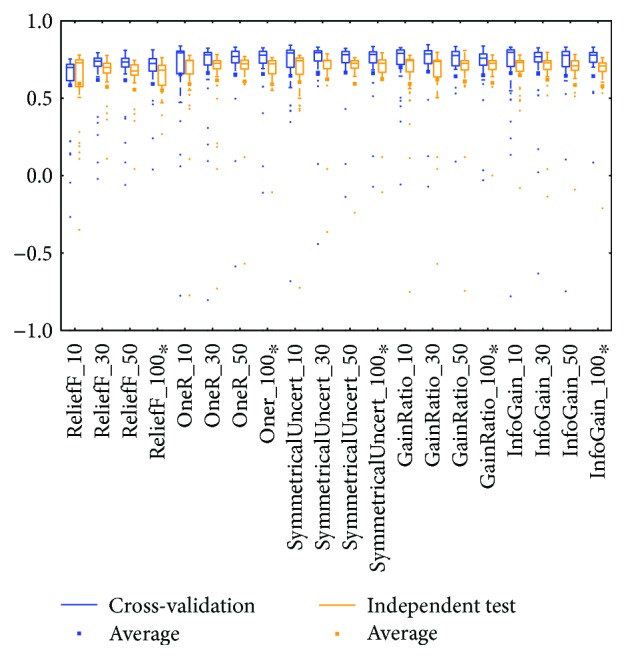
MCC of the BRCA-mRNA group by reduced datasets. ^∗^Note that the subdatasets from OV-miRNA have at most 83 micro-RNAs and thus the scale "100" of OV means 83.

**Figure 4 fig4:**
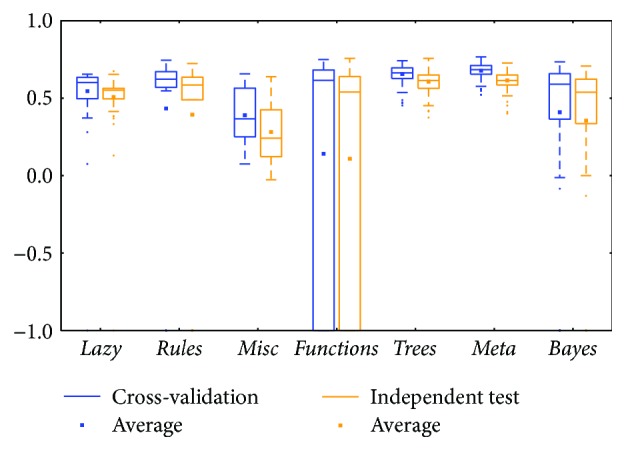
MCC of the BRCA-miRNA group by the *functions* class.

**Figure 5 fig5:**
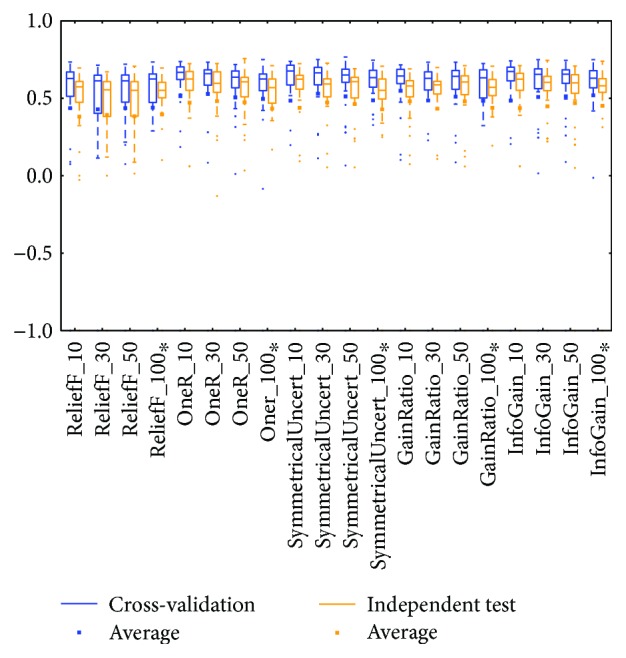
MCC of the BRCA-miRNA group by reduced datasets. ^∗^Note that the subdatasets from OV-miRNA have at most 83 micro-RNAs and thus the scale "100" of OV means 83.

**Figure 6 fig6:**
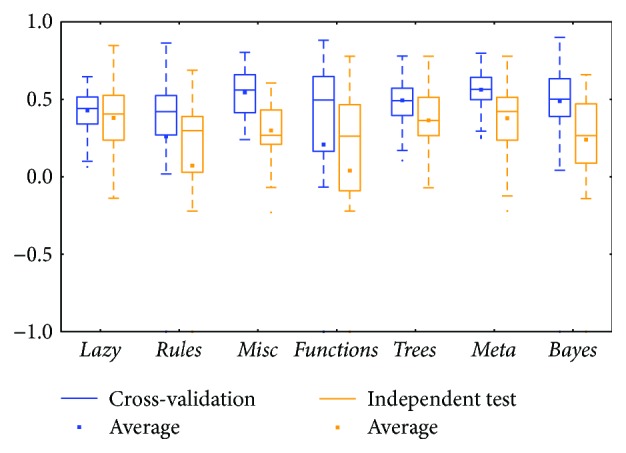
MCC of the KIRC-mRNA group by the *functions* class.

**Figure 7 fig7:**
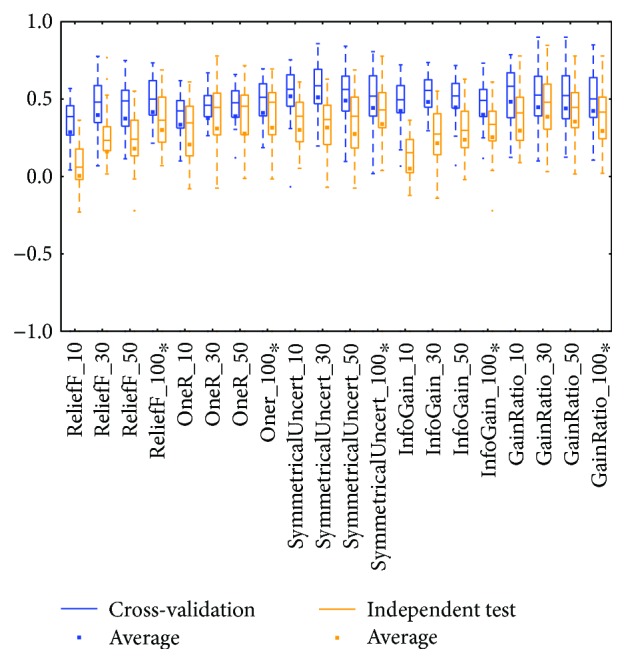
MCC of the KIRC-mRNA group by reduced datasets. ^∗^Note that the subdatasets from OV-miRNA have at most 83 micro-RNAs and thus the scale "100" of OV means 83.

**Figure 8 fig8:**
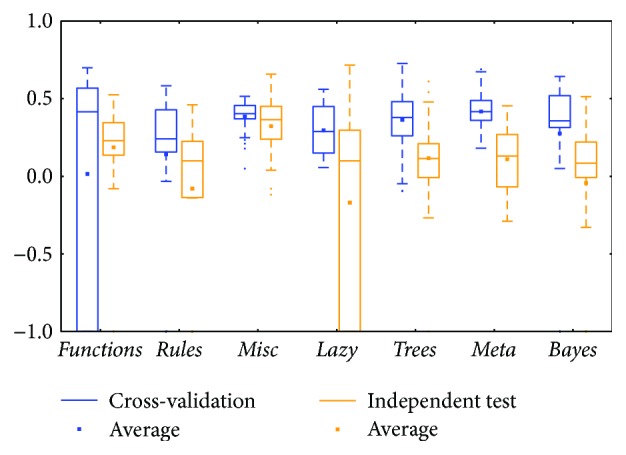
MCC of the KIRC-miRNA group by the *functions* class.

**Figure 9 fig9:**
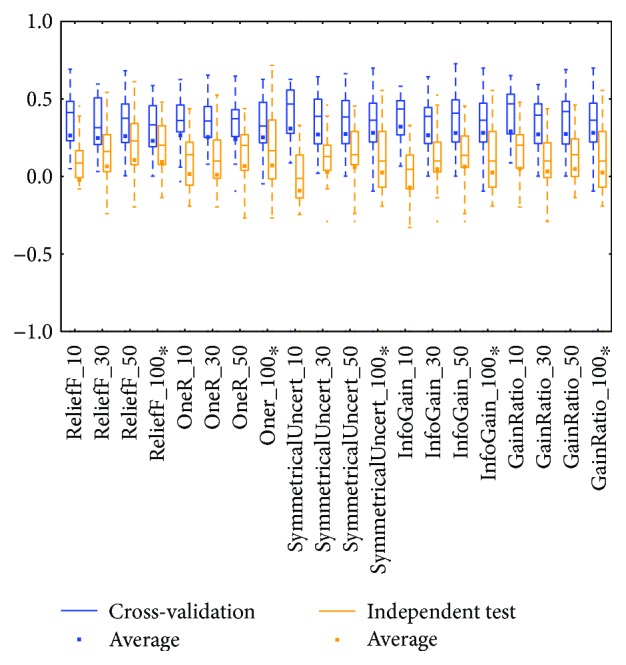
MCC of the KIRC-miRNA group by reduced datasets. ^∗^Note that the subdatasets from OV-miRNA have at most 83 micro-RNAs and thus the scale "100" of OV means 83.

**Figure 10 fig10:**
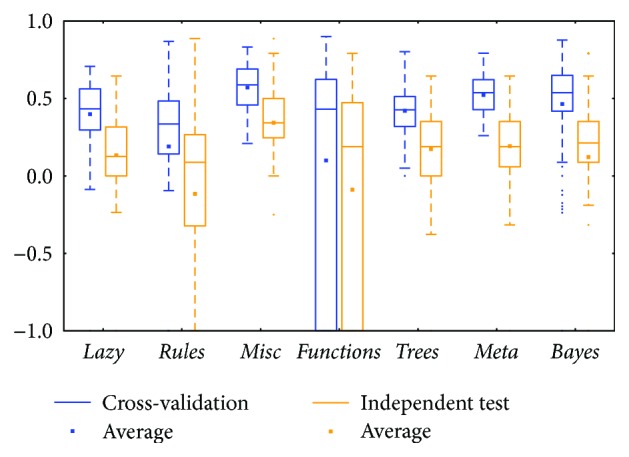
MCC of the OV-mRNA group by the *functions* class.

**Figure 11 fig11:**
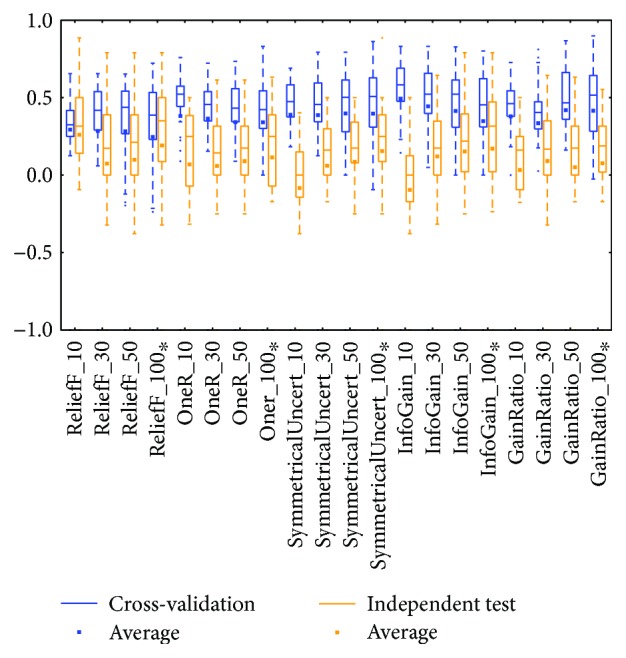
MCC of the OV-mRNA group by reduced datasets. ^∗^Note that the subdatasets from OV-miRNA have at most 83 micro-RNAs and thus the scale "100" of OV means 83.

**Figure 12 fig12:**
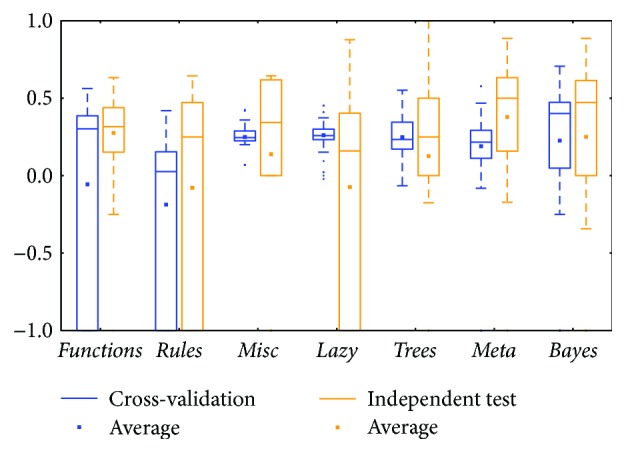
MCC of the OV-miRNA group by the *functions* class.

**Figure 13:**
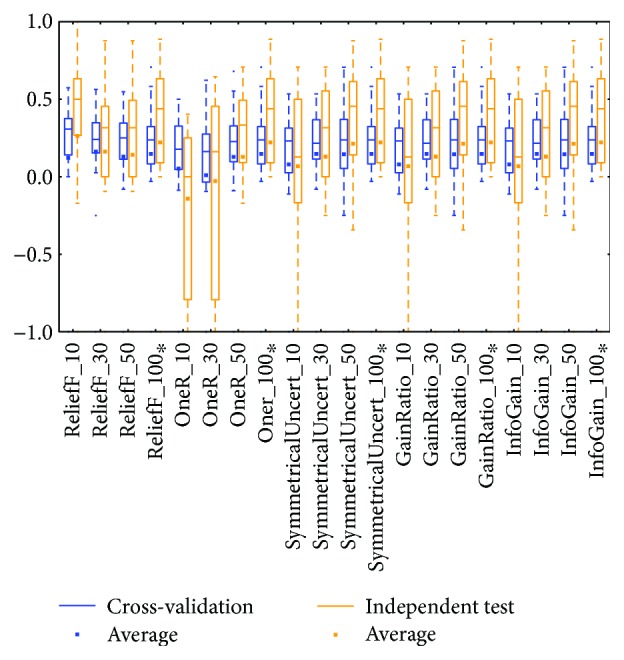
MCC of the OV-miRNA group by reduced datasets. In the 12 box plots, the line in the box is the median. The upper and lower boundaries of the box are Q1 and Q3. The boundaries of the dotted line are the whiskers. ^∗^The subdatasets from OV-miRNA have at most 83 microRNAs, and thus, the scale “100” of OV means 83. ^∗^Note that the subdatasets from OV-miRNA have at most 83 micro-RNAs and thus the scale "100" of OV means 83.

Figure 1 is a flow chart that shows the modeling and integration of the preprocessed datasets. The associated results are listed below. Since there were 5880 modules in total (6 datasets × 980 predictive models per datasets), figures instead of tables were used to present the results (Figures 2 [Fig fig3] [Fig fig4] [Fig fig5] [Fig fig6] [Fig fig7] [Fig fig8] [Fig fig9] [Fig fig10] [Fig fig11] [Fig fig12]–13). The individual cross-validation and independent test results were together listed in the Supplementary file “ModelingResults.xlsx” (available here).

### 3.1. Modeling Results

The prediction performance was ascertained in two ways: by the modeling method class defined by WEKA (see Table 2) and by the different subdatasets generated by the different variable selection methods. Therefore, a total of 3 cancers × 2 sequence methods × 2 kinds of plots = 12 figures were generated for the modeling results.

The *meta* class and *trees* class methods performed better than those in other classes for the two types of BRCA genomics data (mRNA in Figure 2 and miRNA in Figure 4), as evidenced by the best medians and averages. The box for the *trees* class had higher whiskers, but the box for the *meta* class had a smaller IQR.

The KIRC mRNA (Figure 6) and miRNA (Figure 8) datasets showed diverse prediction distributions. In the KIRC-mRNA group, the distributions were similar in the two BRCA groups but the *lazy* class performed in a similar manner in the *meta* and *trees* classes. The distributions were totally different in the KIRC-miRNA class. The *misc* class had the best distribution in the box plot, and the others were far worse. However, most of the medians and averages in the KIRC-miRNA group were less than those in the KIRC-mRNA group.

The situation was similar in the OV-mRNA group (Figure 10) and the OV-miRNA group (Figure 12). The *misc* class was best in the OV-mRNA group datasets, but the *meta* class had relatively better prediction performance for the OV-miRNA group. The *rules* class and *functions* class had very poor distributions in both groups.

Comparing between cross-validation and independent test, most of them were similar; the differences between the whiskers were not huge except for two classes: *functions* and *lazy* (could be found in Figures 2, 8, and 12). The *functions* class often has wider ranges between the two whiskers in cross-validation and is still larger than its independent test. On the contrary, the *lazy* class usually has narrower ranges in cross-validation than its independent test.

Distributions based on different datasets were clearer. In most cases, the dataset (mRNA or microRNA) that contained more attributes had a better distribution (i.e., a smaller IQR or a higher median). However, the peak was sometimes found in a smaller dataset. In more detail, the two BRCA groups (Figures 3 and 5) seemed to be insensitive to the attribute number and the distributions especially medians are very similar. The boxes were larger in the KIRC-miRNA group (Figure 9). The prediction results were sensitive to the attribute number, and the upper whisker increased as the attributes increased, but the lower whisker simultaneously decreased. In comparison, the distributions of the boxes in the KIRC-mRNA group (Figure 7) were better when the attribute number increased to 30 and were relatively stable afterwards. Differently in the OV-mRNA (Figure 11) and the OV-miRNA groups (Figure 13), the boxes contracted when attributes were added.

### 3.2. Voting Results from All Methods

The performance of all of the voted methods is listed in the column “Vote from all methods” of Tables 3, 4, and 5. There is no doubt that the overall voting performance would be lower than optimum, since not all of the voting methods are sufficiently good for the 6 datasets; nevertheless, most of the MCC achieved by voting are better than the average MCC. In addition, a part of the voting performance reached the upper bound (i.e., the maximum), for example, in the *bayes* class in the OV-miRNA group. In more detail, similar to the box plots, the voting performance based on the BRCA datasets was similar and near the upper whisker except for *misc*. The range was larger in the other datasets (e.g., KIRC-mRNA, KIRC-miRNA, OV-mRNA, and OV-miRNA). Different classes in turn, including *bayes*, *functions*, *meta*, *lazy*, *misc*, and *trees*, showed the top three best prediction performance. The *rules* class always ranked lower in the overall voting test, but the *bayes* class showed good voting performance even though its distribution, as indicated by the box plot, was not stable.

### 3.3. Voting Results from Eliminated Methods

The filtering rule was based on the distribution of the prediction results. Values that fell out of the range indicated by the whiskers in a box plot were considered to be outliers. Similarly, in our study, a method with an MCC below the lower whisker was considered as an outlier. There were 5 value selection methods, and each generated 4 subdatasets. If 6 datasets (3 cancers × 2 sequencing techniques) were considered, a modeling method was used: 5 variable selection methods × 4 subdatasets × 6 datasets = 120 times. Therefore, if a method was designated as an outlier more than 6 times (5% of 120), it was not considered in this voting test.

The details of eliminated methods are listed in Table 6. The associated prediction performance was listed in the column “Vote after filtering” in Tables 3, 4, and 5. One or more methods were eliminated in all of the classes except the *meta*. It was evident that *ZeroR* had the most counts, and it was accordingly eliminated. Since only default parameters were used, *LibSVM* and *SPegasos*, which could be considered an optimized *SVM*, were designated as outliers with high counts. Similarly, the *BayesianLogisticRegression* method required parameter optimization and was thus eliminated by many counts. No methods in the *meta* class were eliminated, which indicated that methods based on resampling and an ensemble mechanism were stable and could address varied datasets even they were not always the best.

### 3.4. Voting Results from the Combined Datasets

As mentioned in the Preprocessing, the datasets for one cancer comprised the same samples. Therefore, voting from both mRNA and miRNA datasets is possible. The prediction performance is in the last row of Tables 3, 4, and 5. According to these three tables, the effects of the combination were different in the different classes. No class always benefited from the combination, but the MCCs determined by voting were not worse than the lowest MCC (Tables 4 and 5) and sometimes better than both (Table 3).

## 4. Discussion

In this section, the modeling results will be discussed. Therefore, the discussion is comprehensive, and the gene selection and the modeling results are discussed separately.

### 4.1. Coverage of Selected Genes

Five variable selection methods were used in this study, and different mRNA/miRNA datasets were separately generated. If the subdatasets generated are similar, combining multiple variable selection methods is worthless. Therefore, it is necessary to analyze the contents of the subdatasets and determine the similarity of the datasets. Moreover, the importance of the mRNA was evaluated by using a 3rd party database.

The coverage fraction is the number of the genes which are used more than once of all genes, and accordingly, the formula is

(3) $$\begin{array}{c} Coverage=\frac{NumOfSelectedGenes-NumOfIndependentGenes}{NumOfSelectedGenes}. \end{array}$$

According to Tables 7 and 8, the overall coverage was approximately 40% in the datasets from mRNA. However, the overall coverage of the microRNA datasets was much larger, due to dataset scale limitations. Especially in OV, the finally selected microRNAs were the same because there were only 83 microRNAs; therefore, all of them were selected and ranked in the top 100, and the OV-miRNA datasets must be carefully considered when the predictions are integrated. Conversely, the coverage of the two mRNA subdatasets was not large. According to the statistical results for the mRNA datasets, the frequency of shared mRNA increased as the coverage fraction increased. The shared mRNAs were usually ranked lower by the variable selection methods, which means the most commonly used mRNAs were not recognized as crucial genes that would be ranked higher. The reason might be an insufficient sample number to determine the relationships, since there are many mRNAs. Another probable reason is that the gene expression data could not be directly associated with a disease since cancer is a complex group of diseases, so not only one or a small number of gene are correlated, such as in coexpression [39].

### 4.2. Reliability of the Selected mRNAs

To determine if the shared mRNA is important, the Human Protein Atlas database [40] was used to evaluate its importance. The Human Protein Atlas contains a map from mRNA to tissue generated by an antibody-based approach. The gene reliability is recorded in the database, so that the mRNAs selected in this study could be evaluated by using the database. The evaluation had two steps. First, the fraction of Hits/Total in Table 9 was used to determine the number of selected mRNAs in the dataset. Next, the mRNAs' reliability was verified by using the associated record in the “reliability (IH)” table. An mRNA was considered reliable only if the record was designated as “approved” or “supported.”

Table 9 shows that the fractions of hits in the database were approximately 60%, 55%, and 35% for BRCA, KIRC, and OV, respectively. However, the reliable hits ranged near 70% for the three datasets. The reliabilities based on more than 10 hits were always around 70% no matter which dataset was used. Few differences in reliability were found for the three datasets. The fraction of the reliable mRNAs from OV is relatively larger, and smaller for BRCA. Since the number of records in the Human Protein Atlas is still limited, a more reliable discussion and conclusion must await further analysis based on more samples and records.

The records in the Human Protein Atlas will be updated, and more mRNAs will become available. Therefore, the fraction of hits and the reliability will change accordingly and the shared mRNAs that are currently not recorded in this database might be worthy of study. In addition, the reliabilities of the genes were similar whether a gene was independent or shared, and this indicates that the selected genes were representative for a prognosis but might have a redundant function; therefore, the shared genes are not significantly different from the independent genes in reliability.

### 4.3. Reliability of the Modules

The results from cross-validation could be used as the reference to evaluate the reliability by comparing the results with independent test. As an empirical conclusion, in most cases, the results from cross-validation could be better than an independent test due to various reasons such as the overfitting and batch effects, but if the difference is not too large, the modules could be identified as reliable. Reflected in the figures, except a few classes such as the *functions* class and *laz*y class, the MCC from cross-validation had a relatively better predicting performance (e.g., higher median and average or narrower IQR) than independent test. According to the whiskers, most of the classes had small differences, but there was still a lot of the modules that had a large difference which could be reflected by the outliers. Thus, it is still risky to get the unreliable prediction if we only use the modules which have good performance in cross-validation for predicting. However, since most of the methods were reliable, combining the methods together becomes useful and necessary to reduce the risk.

### 4.4. Distributions of the Predictions

A balanced ratio of positive samples to negative samples is an important factor for prediction. The BCRA datasets had the largest sample numbers; therefore, the IQRs were the smallest, which indicates that the MCCs were concentrated toward the median. The boxes become wider for the KIRC, OV-mRNA, and OV-miRNA datasets. The sample number should be guaranteed before modeling if a stable prediction is to be obtained. However, sometimes, many reasons such as cost, privacy, and difficulty limit the sample number, so it is insufficient to confirm the prediction stability. Such predictions should be considered very carefully because overfitting may have occurred.

A basic way to avoid overfitting is to reduce the attribute number for modeling, and that is why 4 subdatasets (i.e., datasets with 10, 30, 50, and 100 samples) were used for modeling. As shown in the Modeling Results, especially in Figures 9 and 11, more attributes relatively improved the overall prediction performance. However, the improvement was still limited by the sample number. All of the BRCA subdatasets had the relatively smallest IQR compared to the others that had the same attribute number. This limitation might apply not only to modeling but also to many other studies which must use the samples as a template to measure the correlations among samples and genes. For example, in a gene set enrichment analysis, genes should be eliminated by statistical methods such as a *t*-test, fold-change, or FDR. In the analysis, the genes are independent from each other when calculating the correlations and thus the validity of the identified genes is only affected by the sample number. If the sample number is too small (less than 10), the *t*-test result is not reliable.

Limited sample and attribute numbers make a prediction sensitive to the datasets. The various distributions in Figures 2, 4, 6, 8, 10, and 12 indicate that the methods in the *meta* class are relatively stable for prediction, which is reasonable because the methods in the *meta* class use other methods for ensemble learning, so they are not sensitive to different dataset distributions as other types of methods are. The methods in the *trees* class were similar but had a more varied performance than the *meta* class methods because the classification and regression tree algorithms can be as simple as REPTree or as complex as random forest. Therefore, the boxes of the *trees* class usually had a larger IQR than the *meta* class. However, the *misc* class performed best for two datasets, but only two methods were contained in this class. Based on the algorithm, the methods in the *misc* class were much different and thus could have a much more variable performance for different datasets. Except for the *rules* class, the methods in the *bayes* class also had a relatively inferior performance, which might have been caused by the low sample number, because probability-based methods are sensitive on a modeling scale. The performance of methods in the *functions* class was more skewed; the boxes usually had a good upper whisker but a poor lower whisker. One reason for these might be that the algorithms in this class are also different from those in the *misc* class. There were 6 methods in the *functions* class, and thus the prediction performance varied widely. Another reason might be parameter optimization; less parameter optimization would affect all of the methods, but the methods in the *functions* class might be the most affected because they are much more sensitive to the parameters when only default parameters were used.

The high similarity of the gene data used would lead to a similar prediction performance, but when the similarity is lower than 50%, as reflected by the coverage in Table 7 or Table 8, the associated box plots were not significantly similar (as shown in Figures 3, 5, 7, 9, and 11).

According to the coverage of mRNA and microRNA used and shown in Tables 7 and 8, the coverage among the datasets was not large, but most of the medians from different subdatasets for the same cancer were similar, and this is reflected in the boxplots (Figures 3, 5, 7, 9, and 11), which indicates that some of the information contained in the genes was duplicated. In other words, the genes eliminated by the variable selection methods are representative for modeling, but not comprehensive. The duplicated genes could arise from similar genetics or pathology; for example, they could have the same genetic regulation pathway or simply be coexpressed, so that only one would be sufficient for modeling. Additionally, duplication could indicate that variable selection and machine learning methods are not sufficient to find out all of the disease-correlated genes. On the one hand, current machine learning methods can only determine some disease-associated genes, so further study might be necessary. On the other hand, the voting scheme provided in this study could be helpful for evaluating the relationship between cancer and genes.

As previously mentioned, the predictions using different datasets differed, meaning that we cannot determine which method is best for all datasets. The separate use of different modeling methods will result in a loss of information, so using ensemble methods to integrate the modeling results is necessary.

### 4.5. Effects of Voting

A sufficiently large fraction of coverage in the data space is necessary for good voting performance, and the average accuracy must not be too low. Therefore, if only the box plots are considered, the expected performance of the *bayes* class is much lower. However, the result is anomalous in that the *bayes* class showed a good ensemble classification performance (it was ranked in top three) for 5 datasets. On the other hand, the *meta* and *trees* classes were not as good as the *bayes* class even though they had relatively similar distributions. One reason is many ensemble algorithms are contained in those two classes, so that the voting had already been accomplished, so the prediction results were concentrated near the median or the average. It is not difficult to see that in most cases, the voting results of the two classes were near the average. Another reason is that only default parameters were used in the entire test. Many ensemble methods must optimize the submethods for ensemble learning, and the resampling methods also must be optimized.

As a comparison, the voting performance from the eliminated methods was similar. However, the voting results were not always better than the original results. The *bayes* class was negatively affected by the filter. The MCCs based on the *bayes* class were not larger than prior to voting. The *rules* and *functions* classes were benefited by the filter, and the MCCs were improved for most of the datasets. The *trees* class was slightly benefited in the BRCA-miRNA group but was not globally affected as the *meta* class was. The other classes, including the overall voting, were affected positively or negatively by different datasets. The biased effects could indicate that the methods were sensitive to the datasets; the prediction performance of a method changed greatly when the dataset changed. On the other hand, the overall performance was not affected too greatly. One reason was the *meta* class methods, which showed a stable prediction ability, were not eliminated, and thus, the overall results remained stable. Another reason might be that the performance effects were polarized. For example, the *bayes* class that showed the best comprehensive performance was negatively affected but the *rules* class was benefited. Therefore, the overall differences from the two voting mechanisms could be less.

The biased effects mean that a filter might not be that necessary if there is no prior knowledge. However, the voting by combining the mRNA and miRNA datasets could produce better performance if the sample size is sufficiently large, as shown in Table 3. Moreover, as shown in Tables 4 and 5, even the sample size is insufficient, the results will not become worse. Since the methods are not weighted, the results support that most of the methods will produce the same prediction, so combining the two datasets will be beneficial.

## 5. Conclusions

The purpose of this study was to discover a reliable way to predict unknown data to reduce the risk of error prediction when not enough samples were used for modeling. The distribution of the modeling performance indicated that the best methods were different for different datasets; therefore, the methods were integrated using a voting protocol. Finally, we proposed a better way to model different gene expression datasets. In conclusion, no prior knowledge exists; a comparison of the prediction results for three cancers indicates that the methods in the *bayes* class show a good ensemble performance, even though the individual methods are not as stable as those in the *meta* or *trees* classes. The *meta* and *trees* classes already contain many ensemble methods; therefore, their performance is stable but, again, not good for ensemble twice. Therefore, using the methods in the *bayes* class as a group and one of the algorithms in the *meta* class might be a practical approach for a dataset without sufficient prior knowledge. If prior knowledge exists for a cancer, the methods and datasets used can be more specific. For example, this study indicates that using miRNA as an attribute for modeling the OV data could yield a better result than using mRNA, if we knew that at first, some of the negative effects could be avoided. We hope that the scheme can facilitate related studies of genetic data modeling and elucidate important genes to enhance the reliability of the final model.

## Figures and Tables

**Table 1:** Scales of the three datasets.

Disease name	Sample number in modeling dataset	Sample number in independent test dataset	Number of kept genes	
BRCA	558	141	mRNA	24585
			miRNA	722
KIRC	112	29	mRNA	9119
			miRNA	190
OV	66	18	mRNA	4390
			miRNA	83

**Table 2:** Methods used.

Class	Method names
*bayes*	NaiveBayes, BayesianLogisticRegression, BayesNet, ComplementNaiveBayes, DMNBtext, NaiveBayesMultinomial, NaiveBayesMultinomialUpdateable
*functions*	Logistic, MultilayerPerceptron, RBFNetwork, SimpleLogistic, SPegasos, VotedPerceptron, LibSVM
*lazy*	IB1, IBk, KStar, LWL
*meta*	AdaBoostM1, Bagging, Dagging, Decorate, END, FilteredClassifier, LogitBoost, MultiBoostAB, MultiClassClassifier
*misc*	HyperPipes, VFI
*rules*	ConjunctiveRule, DecisionTable, DTNB, NNge, OneR, PART, Ridor, ZeroR
*trees*	ADTree, BFTree, DecisionStump, FT, J48, J48graft, LADTree, LMT, NBTree, RandomForest, RandomTree, REPTree

The names, including the class names, are from WEKA. The results and discussion are based on the classes.

**Table 3:** Voting results of BRCA.

Platforms	Class	Vote from all methods	Vote after filtering	MCC avg	MCC max
BRCA-mRNA	*bayes*	0.7620	0.7620	0.5327	0.7766
	*functions*	0.7252	0.7766	0.3696	0.7938
	*lazy*	0.7620	0.7423	0.6797	0.7793
	*meta*	0.7274	0.7274	0.7295	0.8085
	*misc*	0.5821	0.6466	0.5449	0.7869
	*rules*	0.7274	0.7274	0.5069	0.7967
	*trees*	0.7274	0.7274	0.7207	0.8085
	*overall*	0.7274	0.7274	0.5985	0.8085

BRCA-miRNA	*bayes*	0.7237	0.6895	0.3544	0.7067
	*functions*	0.6733	0.6908	0.1080	0.7566
	*lazy*	0.6214	0.6214	0.5067	0.6736
	*meta*	0.6278	0.6278	0.6142	0.7269
	*misc*	0.3203	0.5773	0.2816	0.6383
	*rules*	0.5990	0.6278	0.3933	0.7234
	*trees*	0.6427	0.6602	0.6066	0.7566
	*overall*	0.6405	0.6908	0.4427	0.7566

BRCA-mRNA and BRCA-miRNA	*bayes*	0.7423	0.7252	0.4436	0.7766
	*functions*	0.6555	0.7915	0.2388	0.7938
	*lazy*	0.7595	0.7595	0.5932	0.7793
	*meta*	0.7595	0.7595	0.6719	0.8085
	*misc*	0.5624	0.6756	0.4133	0.7869
	*rules*	0.7080	0.7746	0.4501	0.7967
	*trees*	0.7595	0.7595	0.6636	0.8085
	*overall*	0.7407	0.7746	0.5206	0.8085

All of the measurements in the tables are MCCs, and the vote after filtering is the MCC based on the eliminated methods. The “avg” is the average of the MCCs.

**Table 4:** Voting results of KIRC.

Platforms	Class	Vote from all methods	Vote after filtering	MCC avg	MCC max
KIRC-mRNA	*bayes*	0.4216	0.3672	0.2401	0.6590
	*functions*	0.6292	0.7162	0.0399	0.7785
	*lazy*	0.4682	0.6292	0.3801	0.8474
	*meta*	0.5261	0.5261	0.3780	0.7785
	*misc*	0.4176	0.4105	0.2991	0.6058
	*rules*	0.4385	0.6110	0.0723	0.6885
	*trees*	0.5421	0.5421	0.3649	0.7785
	*overall*	0.6110	0.5421	0.2535	0.8474

KIRC-miRNA	*bayes*	0.2368	0.1805	−0.0433	0.5131
	*functions*	0.0889	0.0530	−0.1698	0.7162
	*lazy*	0.4371	0.3410	0.1865	0.5249
	*meta*	0.1667	0.1667	0.1105	0.4542
	*misc*	0.4606	0.4795	0.3230	0.6590
	*rules*	0.0889	0.2689	−0.0795	0.4606
	*trees*	0.0530	0.1667	0.1165	0.6110
	*overall*	0.1667	0.1667	0.0323	0.7162

KIRC-mRNA and KIRC-miRNA	*bayes*	0.4795	0.4795	0.0984	0.6590
	*functions*	0.2605	0.6885	−0.0649	0.7785
	*lazy*	0.5514	0.4371	0.2833	0.8474
	*meta*	0.2300	0.2300	0.2442	0.7785
	*misc*	0.4105	0.5131	0.3110	0.6590
	*rules*	0.2605	0.5249	−0.0036	0.6885
	*trees*	0.4371	0.5249	0.2407	0.7785
	*overall*	0.4371	0.5249	0.1429	0.8474

All of the measurements in the tables are MCCs, and the vote after filtering is the MCC based on the eliminated methods. The “avg” is the average of the MCCs.

**Table 5:** Voting results of OV.

Platforms	Class	Vote from all methods	Vote after filtering	MCC avg	MCC max
OV-mRNA	*bayes*	0.4725	0.4725	0.1217	0.7906
	*functions*	0.1250	0.4725	−0.0890	0.7906
	*lazy*	0.3162	0.3162	0.1328	0.6447
	*meta*	0.1890	0.1890	0.1923	0.6447
	*misc*	0.6139	0.7500	0.3433	0.8864
	*rules*	0.1250	0.3162	−0.1160	0.8864
	*trees*	0.1890	0.1890	0.1734	0.6447
	*overall*	0.3162	0.3162	0.0890	0.8864

OV-miRNA	*bayes*	1.0000	0.8771	0.2508	0.8864
	*functions*	0.5000	0.6139	−0.0740	0.8771
	*lazy*	0.6447	0.6447	0.2756	0.6325
	*meta*	0.7559	0.7559	0.3786	0.8864
	*misc*	0.7559	0.6139	0.1384	0.6447
	*rules*	0.3430	0.7559	−0.0783	0.6447
	*trees*	0.5000	0.7559	0.1258	1.0000
	*overall*	0.7559	0.7559	0.1432	1.0000

OV-mRNA And OV-miRNA	*bayes*	0.4725	0.4725	0.1863	0.8864
	*functions*	−1.0000	0.7500	−0.0815	0.8771
	*lazy*	0.7500	0.8771	0.2042	0.6447
	*meta*	0.3162	0.3162	0.2854	0.8864
	*misc*	0.6139	0.7500	0.2408	0.8864
	*rules*	0.3430	0.3162	−0.0971	0.8864
	*trees*	0.3162	0.4725	0.1496	1.0000
	*overall*	0.3162	0.4725	0.1161	1.0000

All of the measurements in the tables are MCCs, and the vote after filtering is the MCC based on the eliminated methods. The “avg” is the average of the MCCs.

**Table 6:** Methods eliminated as outliers.

Class	Names	Counts
*bayes*	BayesianLogisticRegression	78
	DMNBtext	24

*functions*	SPegasos	113
	VotedPerceptron	19
	LibSVM	106

*lazy*	KStar	8

*meta*	*none*	/

*misc*	HyperPipes	12

*rules*	ConjunctiveRule	56
	OneR	9
	ZeroR	120

*trees*	BFTree	14
	DecisionStump	20

The counts are the number of methods whose MCC is lower than the lower whisker in the box plot.

**Table 7:** Coverage of the selected genes from mRNA.

Disease	ShareNum	Subdata scale			
		10	30	50	100
BRCA	5	0	2	2	5
	4	4	5	10	25
	3	4	13	21	45
	2	2	16	26	42
	1	18	49	85	156
	Total	28	85	144	273
	Coverage fraction	35.7%	42.4%	40.1%	42.9%

KIRC	5	0	0	0	2
	4	0	3	3	8
	3	2	6	14	28
	2	6	22	34	71
	1	32	76	128	232
	Total	40	107	179	341
	Coverage fraction	20%	29%	28.5%	32%

OV	5	0	1	2	5
	4	1	2	7	16
	3	1	8	16	55
	2	9	27	34	43
	1	25	59	96	160
	Total	36	97	155	279
	Coverage fraction	30.6%	39.2%	38.1%	42.7%

The coverage fraction is the number of the genes which are used more than once in all of the genes, and accordingly, the formula is Coverage = NumOfSelectedGenes − NumOfIndependentGenes/NumOfSelectedGenes. The ShareNum is the number of a gene used in the subdatasets. For example, in Table 7, the value in the OV-miRNA group with ShareNum 3 and data scale 10 is 1; it means that there is one gene which is used by 3 subdatasets and each subdataset has 10 microRNAs as the attributes.

**Table 8:** Coverage of the datasets from miRNA.

Disease	ShareNum	Subdata scale			
		10	30	50	100^∗^
BRCA	5	1	2	2	8
	4	3	8	17	47
	3	3	11	23	31
	2	3	9	12	26
	1	18	57	79	127
	Total	28	87	133	239
	Coverage fraction	35.7%	34.5%	40.6%	46.9%

KIRC	5	1	14	16	35
	4	3	6	13	53
	3	5	7	19	12
	2	2	7	11	9
	1	14	21	39	59
	Total	25	55	98	168
	Coverage fraction	44%	61.8%	60.2%	64.9%

OV	5	0	5	17	83
	4	3	15	27	0
	3	7	10	6	0
	2	3	5	12	0
	1	11	25	15	0
	Total	24	60	77	83
	Coverage fraction	54.2%	58.3%	80.5%	100%

The coverage fraction means the number of the genes which are used for more than 1 times in all of the genes, and accordingly, the formula is Coverage = NumOfSelectedGenes − NumOfIndependentGenes/NumOfSelectedGenes. The ShareNum is the number of a gene that is used for the subdatasets. For example, in Table 8, the value in the OV-miRNA group with ShareNum 3 and data scale 10 is 7; it means that there are 7 genes which are used by 3 subdatasets and each subdataset has 10 microRNAs as the attributes. ^∗^The subdatasets from the OV-miRNA group have at most 83 microRNAs, and thus, the scale “100” of OV-miRNA means 83.

**Table 9:** Reliability of selected mRNAs.

Names	Data scale	Reliable/Hits/ShareNum			
		10	30	50	100^∗^
BRCA	5	0	1/2/2	1/2/2	4/5/5
	4	4/4/4	4/5/5	8/9/10	13/17/25
	3	0/2/4	2/6/13	5/10/21	13/21/45
	2	0/0/2	5/6/16	8/12/26	18/29/42
	1	8/10/18	27/33/49	39/58/85	68/97/156
	Total	12/16/28	39/52/85	61/91/144	116/169/273
	Hits/Total	57.1%	61.2%	63.2%	61.9%
	Reliable/Hits	75%	75%	67%	68.6%

KIRC	5	0	0	0	0/0/2
	4	0	0/0/3	0/0/3	2/4/8
	3	2/2/2	2/2/6	4/6/14	12/17/28
	2	2/2/6	10/17/22	19/27/34	26/40/71
	1	15/18/32	31/42/76	45/63/128	98/129/232
	Total	19/22/40	43/61/107	68/96/179	138/190/341
	Hits/Total	55%	57%	53.6%	55.7%
	Reliable/Hits	86.4%	70.5%	70.8%	72.6%

OV	5	0	1/1/1	1/1/2	2/2/5
	4	1/1/1	0/1/2	2/3/7	2/4/16
	3	0/0/1	1/1/8	1/2/16	12/16/55
	2	0/2/9	5/8/27	6/10/34	14/18/43
	1	7/8/25	13/21/59	27/34/96	50/64/160
	Total	8/11/36	20/32/97	37/50/155	80/104/279
	Hits/Total	35%	33%	33.3%	37.3%
	Reliable/Hits	72.7	62.5%	74%	76.9%

The table that records the hits and reliability in the “Human Protein Atlas” database. The “ShareNum” is the same in Table 8, and the “Hits” is the number of mRNAs recorded in the “Human Protein Atlas” database. The “Reliable” is the number of reliable hits. The reliability is measured by using the associated record in the “reliability (IH)” table. An mRNA is considered reliable only if the record is “approved” or “supported.” “Hits/Total” and “Reliable/Hits” are calculated simply by using the row “Total” for division. For example, the last two elements in the last column are 37.3% and 76.9%. They are calculated by the element in the associated row in “Total,” such as 80/104/279, where 37.3% = 104/279 and 76.9% = 80/104. ^∗^The subdatasets from the OV-miRNA group have at most 83 microRNAs, and thus, the scale “100” of OV means 83.

## Supplementary Materials

Supplementary Materials As mentioned in Results, a file named “ModelingResults.xlsx” was provided as the supplementary material for recording the 5880 modeling results which were used for generating Figures 2 [Fig fig3] [Fig fig4] [Fig fig5] [Fig fig6] [Fig fig7] [Fig fig8] [Fig fig9] [Fig fig10] [Fig fig11]–12.
